# Aleuritolic Acid Impaired Autophagic Flux and Induced Apoptosis in Hepatocellular Carcinoma HepG2 Cells

**DOI:** 10.3390/molecules23061338

**Published:** 2018-06-02

**Authors:** Hua Yi, Kun Wang, Biaoyan Du, Lina He, Hiuting HO, Maosong Qiu, Yidan Zou, Qiao Li, Junfeng Jin, Yujuan Zhan, Zhongxiang Zhao, Xiaodong Liu

**Affiliations:** 1 Department of Pathology, Guangzhou University of Chinese Medicine, Guangzhou 510006, China; 020693@gzucm.edu.cn (H.Y.); wk1299461695@126.com (K.W.); 020056@gzucm.edu.cn (B.D.); lina_he00@hotmail.com (L.H.); 2 Research Center for Integrative Medicine, Guangzhou University of Chinese Medicine, Guangzhou 510006, China; zouyidan123@outlook.com (Y.Z.); 18813966463@163.com (Q.L.); yujuanzhan@163.com (Y.Z); 3 Department of Anaesthesia and Intensive Care, The Chinese University of Hong Kong, Hong Kong 999077, China; idyhtho@gmail.com; 4 School of Pharmaceutical Sciences, Guangzhou University of Chinese Medicine, Guangzhou 510006, China; qmsfly@outlook.com; 5 Department of Pathology, Zunyi Medical College (Zhuhai Campus), 519000 Zhuhai, China; jinjunfeng01@126.com

**Keywords:** aleuritolic acid, autophagy, apoptosis

## Abstract

Aleuritolic acid (AA) is a triterpene that is isolated from the root of *Croton crassifolius* Geisel. In the present study, the cytotoxic effects of AA on hepatocellular carcinoma cells were evaluated. AA exerted dose- and time-dependent cytotoxicity by inducing mitochondria-dependent apoptosis in the hepatocellular carcinoma cell line, HepG2. Meanwhile, treatment with AA also caused dysregulation of autophagy, as evidenced by enhanced conversion of LC3-I to LC3-II, p62 accumulation, and co-localization of GFP and mCherry-tagged LC3 puncta. Notably, blockage of autophagosome formation by ATG5 knockdown or inhibitors of phosphatidylinositol 3-kinase (3-MA or Ly294002), significantly reversed AA-mediated cytotoxicity. These data indicated that AA retarded the clearance of autophagic cargos, resulting in the production of cytotoxic factors and led to apoptosis in hepatocellular carcinoma cells.

## 1. Introduction

*Croton crassifolius* Geisel (family: Euphorbiaceae) is a medicinal plant widely distributed across Southern China and Asia, including Laos, Thailand, and Vietnam [1]. The root of *C. crassifolius* is used as a traditional Chinese medicine to treat snake bites, pain, pharyngitis, jaundice, rheumatoid arthritis, and other ailments [2]. *C. crassifolius* is also used by indigenous populations in Thailand to treat tumors [3]. Indeed, a variety of compounds with cytotoxic activity has been isolated from *C. crassifolius* by Tian et al. [4]. Recently, we have isolated several triterpenes, including aleuritolic acid (AA), from the root of *C. crassifolius*. Although these compounds were not novel triterpenes, no pharmacological activity was reported. We asked whether AA could exert anti-tumor like actions and represent one of the active ingredients in *C. crassifolius*.

As *C. crassifolius* is used to treat liver-related diseases in traditional Chinese medicine, we selected the human hepatocellular carcinoma (HepG2) cell line as a model to screen the cytotoxic activity of compounds extracted from *C. crassifolius*. Here, we report a novel finding of the potent anticancer activity of AA in hepatic cancer that is likely related to autophagy, as evidenced by morphological changes and molecular evaluation of HepG2 cells treated with AA.

Autophagy is a self-degradative pathway involving the removal of damaged or superfluous proteins and organelles [5]. While autophagy can prevent tumorigenesis in some contexts [6], it can also facilitate tumor cell survival and promote tumor growth, thus affecting the efficacy of cancer therapies [7]. The overall activity of the autophagy pathway can be robustly measured by autophagic flux, which describes the rate of autophagosome-lysosome fusion and subsequent degradation of the intra-autolysosomal contents. Lysosomal acidification is crucial for the degradation of autophagic cargo because the luminal enzymes optimally function in an acidic environment. González-Rodríguez et al. reported that impaired autophagic flux caused apoptosis in hepatocytes [8]. Additionally, defective lysosomal acidification impairs autophagic flux [9]. Inhibition of autophagic flux has recently been reported as a novel tumor treatment strategy [10]. In addition, suppression of autophagosome-lysosome fusion sensitized human cancer cells to cisplatin-induced apoptosis [11].

In this article, we studied the anticancer activity and the underlying mechanism of aleuritolic acid in hepatic cancer.

## 2. Results

### 2.1. Cytotoxic Activity of AA In Vitro

**Figure 1:**
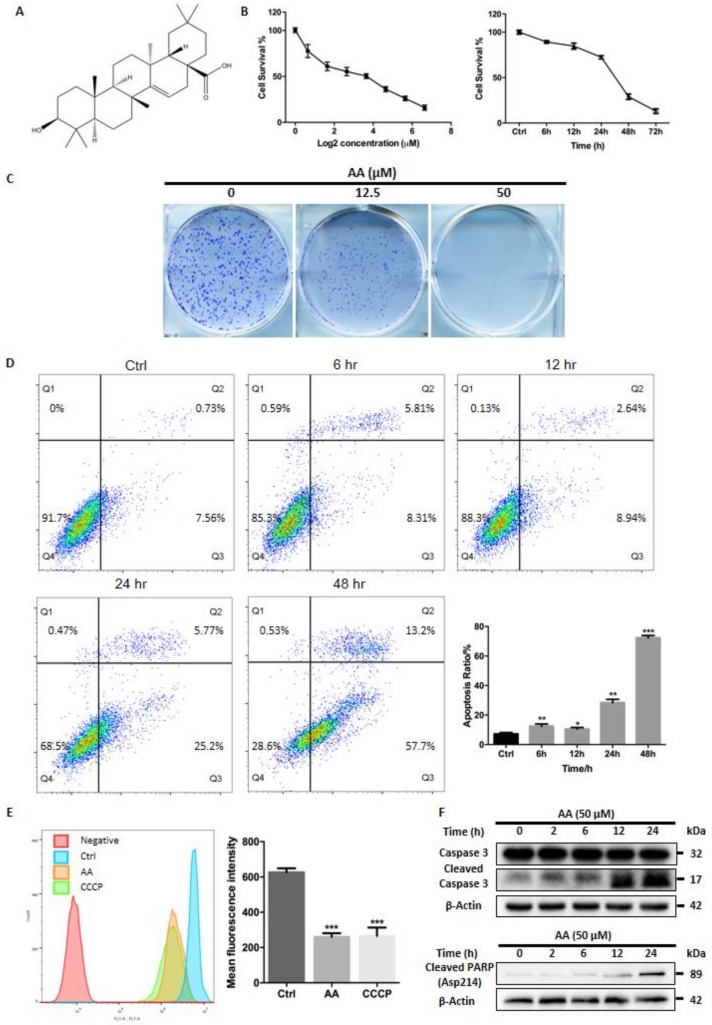
AA exhibited cytotoxic effects against HepG2 cells. (**A**) The molecular structure of aleuritolic acid is shown. (**B**) MTT assay shows that AA caused dose-dependent and time-dependent inhibitory effects on growth of HepG2 cells. The IC50 is 10.2 μM. (**C**) Colony formation assays demonstrated a dose-dependent inhibitory effect of AA on colony formation of HepG2 cells. (**D**) AA treatment for different times induced early and late apoptosis in HepG2 cells. * *p* < 0.05, ** *p* < 0.01, *** *p* < 0.001, One-way ANOVA. (**E**) AA treatment depolarized mitochondria in HepG2 cells. The effect was comparable with CCCP, an uncoupler of mitochondrial respiration. *** *p* < 0.001, One-way ANOVA. (**F**) AA treatment caused a time-dependent accumulation of cleaved caspase-3 and cleaved PARP (Asp214).

The chemical structure of AA was determined with NMR (Supplementary Materials—NMR) and shown in the Figure 1A. MTT assays were performed to assess the cytotoxicity of AA on hepatocellular carcinoma cells. HepG2 cells were treated with different concentrations of AA (100, 50, 25, 12.5, 6.25, 3.125, 1.5625, and 0 μM) for 24 h. AA inhibited growth of HepG2 cells in a dose- and time-dependent manner (Figure 1B), and the IC50 was found to be 10.2 μM. Similarly, colony formation assays demonstrated a reduction in colony formation of HepG2 cells treated with AA (Figure 1C), and a concentration as low as 12.5 μM of AA greatly reduced both the number and size of the colonies formed. Higher concentrations of AA (50 μM) completely blocked HepG2 cell colony formation. Assessment of apoptosis by flow cytometry demonstrated that treatment with AA for different time increased early apoptosis (ratio of Annexin V positive/PI negative cells for 0, 6, 12, 24 and 48 h were 6.06 ± 1.30%, 6.46 ± 1.60%, 7.05 ± 1.69%, 21.67 ± 3.06%, and 56.43 ± 1.36%, respectively) and late apoptosis (ratio of Annexin V/PI double positive cells for 0, 6, 12, 24 and 48 h were 1.20 ± 0.45%, 6.07 ± 0.52%, 3.44 ± 0.70%, 6.79 ± 0.93%, and 15.90 ± 2.34%, respectively) in HepG2 cells (Figure 1D). As expected, AA (50 μM) also abolished mitochondrial membrane potential with a similar extent of CCCP (10 μM) treatment. Compared with vehicle, AA treatment caused a 58.5 ± 3.3% reduction in mean fluorescence intensity (MFI), while CCCP reduced MFI by 57.6 ± 7.6% (Figure 1E). Finally, AA treatment induced a time-dependent accumulation of cleaved caspase-3 and cleaved PARP (Asp214), a well-known marker of apoptosis (Figure 1E). These data suggested that AA exerted cytotoxic activity in HepG2 cells by inducing apoptosis.

### 2.2. Treatment with AA Impairs Autophagic Flux in HepG2 Cells

**Figure 2:**
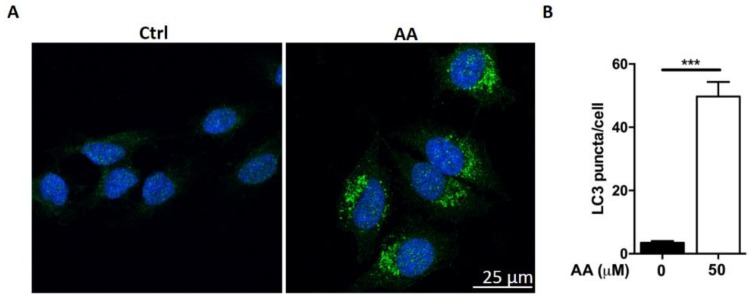

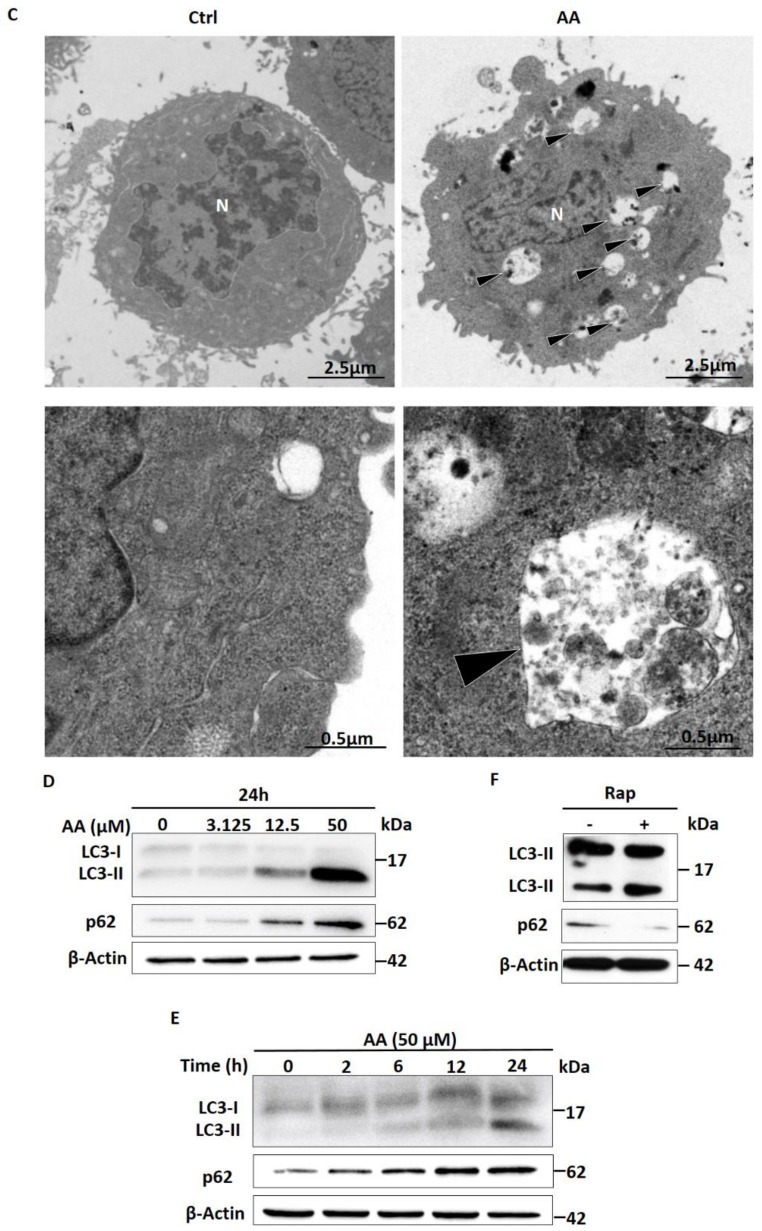
AA induced autophagy dysregulation in HepG2 cells. (**A**, **B**) A large number of LC3 positive puncta (mean = 50, *n* = 54) are seen after AA treatment. In contrast, fewer than 10 LC3 puncta (mean = 3, *n* = 13) are observed in control cells. Student’s *t*-test *p*-value: *** *p* < 0.001. (**C**) AA induces the accumulation of vacuole-like structures in the cytoplasm (arrow head), while few vacuoles are observed in DMSO-treated cells. In the lower panel, higher magnification images show that AA-induced vacuoles contained cellular organelles (arrow head). (**D**,**E**) AA treatment causes p62 accumulation and conversion of LC3 I to LC3II in a time- and dose-dependent manner. (**F**) Rapamycin treatment leads to p62 degradation and conversion of LC3 I to LC3II in HepG2 cells.

**Figure 3:**
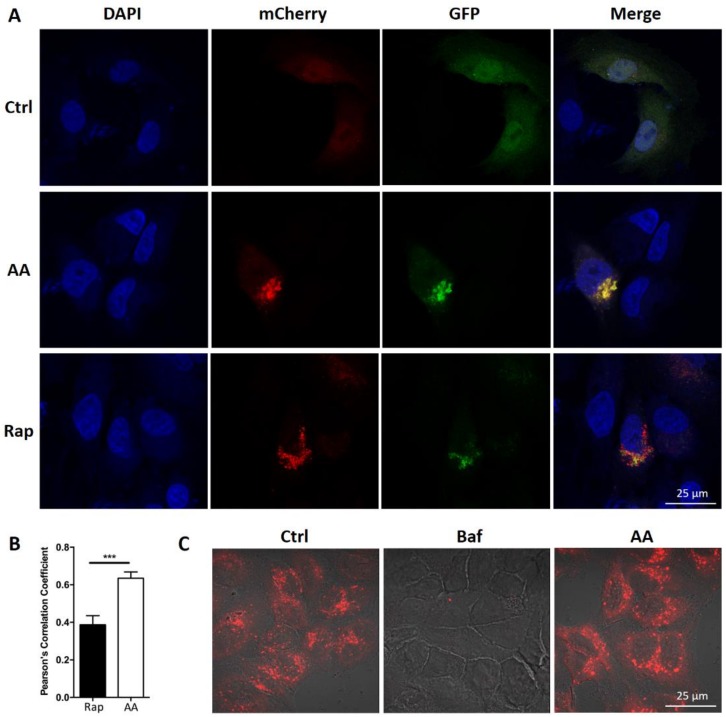
AA impaired autophagic influx. (**A**,**B**) Red LC3 puncta are greatly induced in cells after treatment with AA or rapamycin. Co-localized green fluorescence is significantly increased in AA-treated cells (*n* = 20) as compared with rapamycin-treated cells (*n* = 20). Student’s *t* test *p*-value: *** *p* < 0.01. (**C**) AA did not affect the fluorescent signals of lysotrackers. In contrast, Bafilomycin A1, the V-ATPase inhibitor completely abolishes the fluorescence.

We observed that AA treatment induced the formation of vacuoles in HepG2 cells (data not shown). We queried whether treatment with AA affects autophagic flux in HepG2 cells. Cells were stained with anti-LC3 antibody. Many LC3 positive puncta (mean = 50, *n* = 54) were observed after AA treatment in HepG2 cells (Figure 2A,B). In contrast, less than 10 LC3 puncta (mean = 3, *n* = 13) were observed in control cells. We also evaluated cellular and organelle morphology with a TEM assay. It showed that AA treatment induced the accumulation of vacuole-like structures in the cytoplasm, while few vacuoles were observed in DMSO (vehicle)-treated cells (Figure 2C, arrow head). Higher magnification revealed that the vacuoles induced by AA treatment contained cellular organelles (Figure 2C, arrow head), suggesting that AA treatment induced macroautophagy. Furthermore, Western blot assessment showed that the conversion of LC3-I to LC3-II induced by AA treatment occurred in a time- and dose-dependent fashion (Figure 2D,E). These observations were consistent with those following treatment with rapamycin, a well-known inducer of autophagy. These data indicated that AA treatment modulates autophagic flux. Interestingly, rapamycin treatment led to p62 degradation (Figure 2F), whereas AA caused p62 accumulation in HepG2 cells (Figure 2D,E). p62 functions as a receptor for cargo that is degraded by autophagy. Upon autophagy induction, p62, per se, is also degraded in the autolysosome. In contrast, autophagy inhibitors cause the accumulation of p62. Our observation therefore indicated that AA treatment might lead to impairment of the autophagic flux. We performed mCherry-GFP-LC3 reporter assay to assess autolysosome function. As expected, red LC3 puncta were significantly induced in HepG2 cells after treatment with AA or rapamycin. However, co-localized green fluorescence was significantly increased in cells treated with AA compared to cells treated with rapamycin (Figure 3A,B). Interestingly, while Bafilomycin A1 (V-ATPase inhibitor) treatment completely abolished lysotracker-emitting fluorescence, AA (50 μM) had no effects on the fluorescent intensity (Figure 3C). Together with p62 accumulation, these results demonstrated that AA might impair autophagic flux in HepG2 cells. However, this action was unlikely mediated by interrupting lysosomal acidification. 

### 2.3. Impaired Autophagic Flux Contributes to AA Induced HepG2 Cell Death

**Figure 4:**
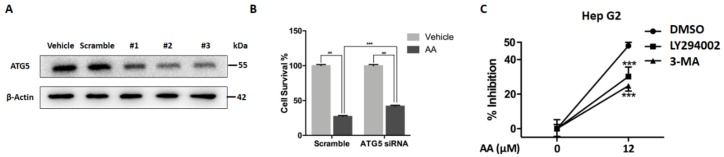
Autophagosome formation contributes to AA induced cytotoxicity. (**A**) ATG5-specific siRNA oligos reduced ATG5 protein levels in HepG2 cells. The third (#3) siRNA oligos was selected for subsequent experiments. (**B**) Atg5 knockdown significantly reverses the inhibitory effects of AA on HepG2 cells, ### *p* < 0.001, *** *p* < 0.001, Two-way ANOVA. (**C**) Pretreatment with 3-MA or LY294002 significantly reduces AA-induced cell death in HepG2 cells.

We next investigated whether impairment of autophagic flux contributed to AA-induced cell death. Atg5 is required for autophagosome formation, and deletion of Atg5 causes autophagy deficiency. Deletion of Atg5 may therefore abolish stress-induced formation of dysfunctional autolysosomes and may prevent cell death. We evaluated the response to AA in HepG2 cells with or without ATG5 knockdown. Three different ATG5-specific siRNA oligos were transfected and all greatly reduced ATG5 protein levels as compared with scramble control (Figure 4A). The third (#3 siRNA) was selected and applied for the cytotoxicity assay. Intriguingly, ATG5 knockdown significantly reversed the inhibitory effects of AA compared with wide type cells (Figure 4B). LY294002 and 3-MA are widely used pharmacological inhibitors of the PI3K pathway that can block autophagosome formation. Consistent with the effects of Atg5 knockdown, pretreatment of HepG2 cells with 3-MA or LY294002 to block autophagosome formation significantly reduced AA-induced cell death (Figure 4C). These data suggested that modulation of autophagy played an important role in AA-induced cell death.

## 3. Materials and Methods

### 3.1. Reagents and Antibodies

Aleuritolic acid (AA) was isolated from *Croton crassifolius* Geisel in our lab, with a purity of >98% (Figure 1A and Supplementary Materials). AA was dissolved in dimethyl sulfoxide (DMSO) to make a 100 mM stock solution; this stock solution was diluted with culture medium before use. Rapamycin was obtained from Tocris Bioscience (Bristol, BS, UK). Anti-MAP1LC3B (GTX127375) and anti-SQSTM1/p62 (GTX100685) were purchased from GeneTex (Hsinchu City, Taiwan). Primary antibodies against Caspase-3 (#9662), cleaved PARP (#5625), β-actin (#4970), and secondary antibodies (HRP linked anti-mouse, HRP linked anti-rabbit, and Alexa Fluor^TM^ 488 conjugated anti-rabbit secondary antibodies) were purchased from Cell Signaling Technology (Danvers, MA, USA). The pBABE-puro mCherry-EGFP-LC3B plasmid was a gift from Jayanta Debnath [12] (Addgene plasmid #22418). The plasmid transfection reagent, polyethylenimine HCl MAX, Linear Mw 40,000 (PEI MAX 40000, #24765) was purchased from Polysciences (Warrington, PA, USA). Human ATG5 siRNA kit containing riboFECTtm CP reagent (for transfection), scramble control, #1, #2, and #3 ATG5 specific siRNA were ordered from Ruibo (Guangzhou, China). The Annexin V-FITC apoptosis detection kit was obtained from Dojindo (Shanghai, China). ProLong Diamond Antifade mounting reagent with DAPI (#P36971), protease inhibitor tablets (#88266) and Pierce BCA protein assay kit (#23227) were purchased from ThermoFisher Scientific (San Jose, CA, USA).

### 3.2. Cell Culture

The HepG2 hepatocellular carcinoma cell line (ATCC HB^®^-8065TM) was purchased from American Type Culture Collection (Manassas, VA, USA). Cells were maintained in high glucose Dulbecco’s modified Eagle’s medium (GibcoTM, #11965118) supplemented with 10% fetal bovine serum (GibcoTM, #10082147) and 1% penicillin/streptomycin (GibcoTM, #15070063) in a humidified incubator at 37 °C and 5% CO_2_.

### 3.3. Cell Viability Assay

Cells were seeded into six replicates in 96-well plates at a density of 3000 cells/well and cultured for 24 h. Cells were then treated with AA or vehicle at the indicated concentrations (see results) for 24 h. After treatment, 10 μL of MTT (3-(4,5-dimethylthiazolyl-2)-2,5-diphenyltetrazolium bromide, 5 mg/mL) reagent was added into each well, and cells were incubated for an additional 2 h at 37 °C. The supernatant was carefully removed, and the remaining formazan crystals were dissolved in DMSO. The absorbance of each well was measured at a wavelength of 570 nm using a microtiter plate reader.

### 3.4. Mitochondrial Membrane Potential Detection

3,3′-Dihexyloxacarbocyanine iodide (DiOC6(3)) was purchased from Thermofisher Scientific and applied to monitor mitochondrial membrane potential according to the manufacturer’s instructions (San Jose, CA, USA). Cells were seeded into six-well plates and treated with vehicle or AA for 24 h. Carbonyl cyanide 3-chlorophenylhydrazone (CCCP) was also applied as a positive control, except that cells were harvested after brief exposure, i.e., 6 h. Cells were trypsinized and washed with PBS two times, followed by DiOC6(3) staining for 20 min in 37 °C water bath. Then cells were washed with PBS for an additional three times, re-suspended with HBSS, and submitted to flow cytometry for analysis (Becton-Dickinson, Franklin Lakes, NJ, USA). 

### 3.5. Annexin V/Propidium Iodide Apoptosis Assay

HepG2 cells were seeded in 12-well plates at a density of 1 × 10^5^ cells/well. Cells were treated with vehicle or AA for 24 h. Cells were then dissociated from culture plates and harvested for Annexin V and PI staining, according to the manufacturer’s instructions. Apoptosis was measured by flow cytometry (Becton-Dickinson, Franklin Lakes, NJ, USA).

### 3.6. Transmission Electron Microscopy (TEM)

HepG2 cells were plated into 12-well plates at a density of 1 × 10^5^ cells/well. Cells were treated with vehicle or AA for 24 h. After treatment, cells were harvested and fixed in 2.5% glutaraldehyde overnight, and then incubated with osmium tetraoxide for two hours at 4 °C. Specimens were embedded in epoxy resin. Sections of 100 nm-thickness were prepared and stained with uranyl acetate and lead citrate. Sections were imaged on a Hitachi HT7700 transmission electron microscope (Tokyo, Japan).

### 3.7. LC3 Staining

HepG2 cells were seeded onto glass coverslips in a 12-well plate and treated with vehicle or AA for 24 h. After treatment, cells were fixed with 4% paraformaldehyde (PFA) for 10 min then rinsed in PBS three times for five minutes. Cells were blocked in antibody dilution buffer containing 5% FBS and 0.3% Triton X-100 in PBS for 1 h at room temperature. After blocking, cells were incubated with anti-LC3B (1:1000 dilution; GeneTex) overnight at 4 °C. Cells were then rinsed with PBS three times for five minutes and blotted with Alexa Fluor^TM^ 488-conjugated anti-rabbit secondary antibody for 1 h at room temperature. Cells were rinsed in PBS three times for five minutes before mounting in ProLong Diamond Antifade mounting medium with DAPI. Cells were imaged on a Leica TCS SP8 confocal laser scanning microscopy platform (Wetzlar, Germany). The LC3 puncta in each cell were calculated by a researcher who was blinded to the sample identity.

### 3.8. Lysotracker Staining

LysoTracker Red DND-99 was obtained from ThermoFisher Scientific (San Jose, CA, USA). Cells were seeded and treated similarly as the LC3 staining. The V-ATPase inhibitor, Baflimycin A1 was adopted as a positive control to abolish Lysotracker fluorescence. After treatment, cells were refilled with fresh culture medium with Lysotracker dye (7 nM) and incubated at 37 °C for 15 min, following with three washes of HBSS. The fluorescent pictures were then taken on a Leica TCS SP8 confocal laser scanning microscopy platform. 

### 3.9. Transfection

For transfection with the mCherry-GFP-LC3 vector, cells were plated onto glass coverslips (4 × 10^4^ cells/well). On the day of transfection, the cell culture medium in each well was replaced with DMEM lacking serum and antibiotics (900 μL/well). Plasmid (1 μg) and transfection reagent (PEI; 3 μL) were separately diluted in 50 μL of DMEM lacking serum and antibiotics. Diluted plasmid and PEI were mixed, vortexed briefly, incubated at room temperature for 20 min, and then added to cells. After six hours incubation, the media in each well was replaced with DMEM containing 10% FBS and antibiotics. siRNA transfections were performed according to the manufacturer’s instructions. siRNA stock solution (20 µM) were prepared with RNase free water. For 24-well plates, 2.5 µL of stock and 3 µL of riboFECTtm CP Reagent were mixed and used for transfection. After 24 h, total proteins were extracted to detect the efficiency of knockdown. For 96-well plates, 0.5 µL of stock and 0.6 µL of transfection reagent were applied in each well. 

### 3.10. Western Blotting

Total protein was extracted with cell lysis buffer (50 mM Tris, 150 mM NaCl, 1% NP40, 1 mM EDTA, pH 7.6) containing a cocktail of protease inhibitors. Protein concentration was determined using a Pierce BCA protein assay kit, according to the manufacturer’s instructions (San Jose, CA, USA). Samples (30 µg protein/lane) were separated on a 10% (PARP, β-actin and p62) or 15% (LC3) SDS-polyacrylamide gel, then transferred onto PVDF membranes (0.22 μm pore, Roche, Rotkreuz, Switzerland). After blocking with TBST buffer (20 mM Tris, 137 mM NaCl, 0.1% Tween-20, Ph 8.0) containing 5% non-fat milk, and membranes were incubated with primary antibody against cleaved PARP (1:1000 dilution), p62 (1:1000), LC3 (1:1000), and β-actin (1:3000) overnight at 4 °C. Then membranes were incubated with secondary antibody (1:3000) for 1 h at room temperature. The protein bands were visualized using Immobilon Western Chemiluminescent HRP substrate (Millipore, Burlington, MA, USA).

## 4. Discussion

Autophagy is an essential process that is required for cells to maintain homeostasis. It can serve as a protective mechanism to get rid of superfluous or damaged cellular constituents [13]. Upon induction of autophagy, autophagosome is formed and sequesters cellular waste. The outer membrane of the autophagosome fuses with the lysosomal membrane and forms an autolysosome. Subsequently, the cargos are exposed to the hydrolases and digested in the acidic environment. These degradation products are then released to the cytoplasm for biosynthetic processes or energy generation in cells [14]. Recent studies revealed that autophagosomes that failed to fuse with lysosomes might result in autophagosome accumulation, leading to excessive intracellular ROS and subsequent cell death [15]. Moreover, it has been reported that a higher basal level of autophagy was observed in several types of tumor cells, especially intra-tumor cells. Inhibition of autophagy may, therefore, offer potential interventions to cancer.

In the current study, we reported that AA was a novel autophagy inhibitor and impairment of autophagic flux by AA treatment caused apoptotic cell death in HepG2 cells.

We demonstrated that AA treatment induced dose- and time-dependent cytotoxicity in HepG2 cells (Figure 1B,C). Annexin V/PI assay indicated that cytotoxicity by AA was mainly mediated by apoptosis induction (Figure 1D). Moreover, AA also caused a dramatic loss of mitochondrial membrane potential and accumulation of cleaved caspase-3/cleaved PARP (Figure 1E,F). We concluded that AA exerted cytotoxicity by activating the mitochondrial apoptosis pathway.

Notably, AA induced the accumulation of vesicle-like structures in the early stage of treatment. We asked whether AA might affect autophagy. Indeed, LC3 positive puncta were significantly increased in AA-treated cells than in control cells (Figure 2A,B). TEM assay also showed that cellular organelles could be observed in these vesicles (Figure 2C). Moreover, the LC3-I to LC3-II conversion was significantly increased by AA treatment than vehicle control, in both a time- and dose-dependent manner. However, AA up-regulated p62 level in HepG2 cells, while rapamycin, a well-known autophagy inducer, led to p62 degradation. p62 acts as a cargo receptor or adaptor for autophagic degradation and is subsequently degraded by lysosomal enzymes [16]. In contrast, p62 is accumulated when autophagic flux is interrupted [17]. In fact, the accumulation of p62 has been widely used as an indicator of impaired autophagic flux [18]. To provide a more detailed evaluation of impaired autophagic flux, we performed mCherry-GFP-LC3 plasmid transfection assay [18]. The mCherry-GFP-LC3 construct is a tandem fluorescent-tagged protein and is a useful tool to study autolysosome function. In the acidic environment found in normal autolysosomes, GFP fluorescence is greatly suppressed, while mCherry fluorescence is preserved. An accumulation of yellow (green/red co-localized) florescence indicates impaired autophagic flux with either a dysfunction of autolysosomes or a failure of autophagosome-lysosome fusion. As expected, red LC3 puncta were substantially induced in cells treated with both AA and rapamycin. However, co-localized green fluorescence (acid sensitive) in AA-treated cells was significantly increased, as compared with rapamycin-treated cells, indicating that AA might impair autophagic flux in HepG2 cells (Figure 3A,B). To explore the potential mechanism of autophagic impairment, Lysotracker was applied to evaluate the alterations of acidic organelles. As expected, Bafilomycin A1, which is a specific inhibitor of vacuolar-type proton pumps on lysosomes, completely abolished Lysotracker-emitting fluorescence. In contrast, AA did not affect the fluorescent intensity at the concentration of autophagy impairment and apoptosis induction (Figure 3C). Taken together, we concluded that AA was a novel autophagy inhibitor. The affected phase was unlikely to be autophagosome formation or lysosomal acidification. Instead AA might interrupt autophagosome-lysosome fusion. 

It should be noted that the increase of LC3I to II conversion and p62 level by AA treatment appeared to be earlier than caspase-3 and PARP cleavage (2 h vs. 6 h). This result indicated that autophagy impairment by AA contributed to apoptosis induction later on, as AA most likely affects autophagosome-lysosome fusion. We abolished autophagosome formation by applying ATG5-specific siRNA or chemical inhibitors. As shown in Figure 4B, ATG5 knockdown partially, but significantly, reversed AA-induced cytotoxicity. Similar outcomes were observed when we applied 3-MA and LY294002. Both inhibitors could block PI3K activity and, thus, block autophagosome formation. Pretreatment with 3-MA or LY294002 significantly reduced AA-induced cell death in HepG2 cells. Our finding suggested that AA-induced failure in delivering “packaged” waste (e.g., damaged cellular organelles, Figure 2C) to lysosomes might produce “death-causing effectors”. Nonetheless, efforts to determine the “death-causing effectors” are still required in the future, as ROS are unlikely to be such factors (Supplementary Materials). 

## Supplementary Materials

The following are available online.
