# Supplementary material for: Aleuritolic Acid Impaired Autophagic Flux and Induced Apoptosis in Hepatocellular Carcinoma HepG2 Cells
Source: Molecules. 2018 Jun 2;23(6):1338. doi: 10.3390/molecules23061338 (PMC6100546; doi:10.3390/molecules23061338)
Supplement: Supplementary file 1 [file molecules-23-01338-s001.zip › supplementary materials-NMR.pdf]

Extraction and Isolation

The dried and powdered roots of Croton crassifolius (29.4 kg) were percolated with MeOH (364 L) at room temperature. The extracts were combined and concentrated under reduced pressure to leave a residue, which was then mixed with diatomite and eluted with petroleum ether (60 L), CHCl3 (80 L), and EtOAc (40 L). The petroleum ether extract (1321.4 g) was chromatographed on silica gel column with gradient mixtures of petroleum ether-EtOAc (100:1 → 1:1) to afford ten fractions, Fr. A to Fr. J. Fr. G (2.1 g) was subjected to silica gel column (petroleum ether-acetone, 15:1→ 2:1), then separated over ODS column chromatography with 60% MeOH-H2O to yield compound A (6 mg).

Structure elucidation

Compound A (C30H48O3), white amorphous powder; 1H NMR (CDCl3, 400 MHz) δH: 5.53 (1H, dd, J = 7.8, 3.3 Hz), 0.96 (3H, s), 0.95 (3H, s), 0.93 (3H, s), 0.92 (3H, s), 0.92 (3H, s), 0.91 (3H, s), 0.80 (3H, s) (Supplementary Figure 1A); 13C NMR (CDCl3, 100 MHz) δC: 37.9 (C-1), 27.3 (C-2), 79.1 (C-3), 39.2 (C-4), 55.6 (C-5), 19.0 (C-6), 41.1 (C-7), 38.9 (C-8), 49.3 (C-9), 38.2 (C-10), 17.5 (C-11), 33.8 (C-12), 37.5 (C-13), 160.8 (C-14), 116.9 (C-15), 31.6 (C-16), 51.5 (C-17), 41.6 (C-18), 35.5 (C-19), 29.4 (C-20), 33.5 (C-21), 30.9 (C-22), 28.1 (C-23), 15.7 (C-24), 15.6 (C-25), 26.3 (C-26), 22.6 (C-27), 183.4 (C-28), 32.1 (C-29), 28.8 (C-30) (Supplementary Figure 1B). The spectral data were similar to aleuritolic acid reported previously [1]. Compound A was established as aleuritolic acid.

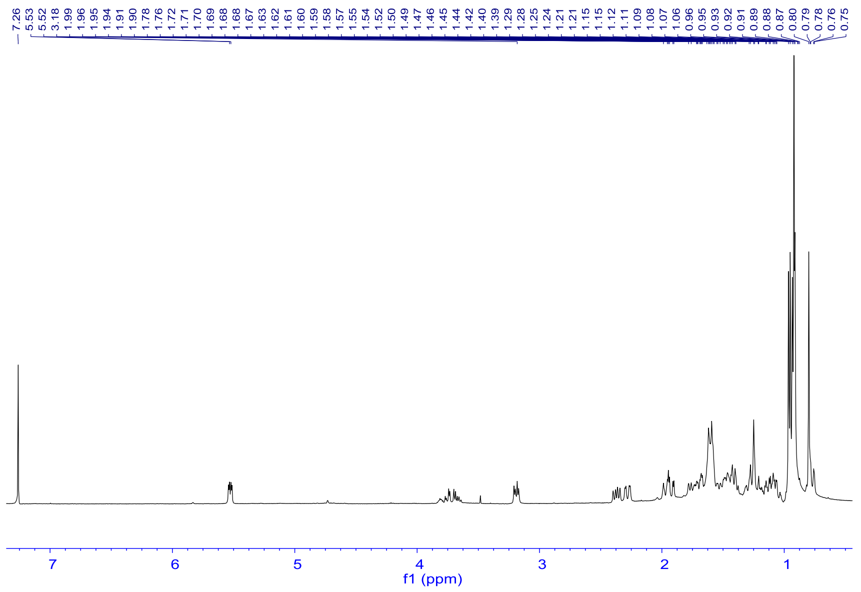

Supplementary Figure 1 1H NMR spectrum of Compound A (400 MHz, CDCl3)

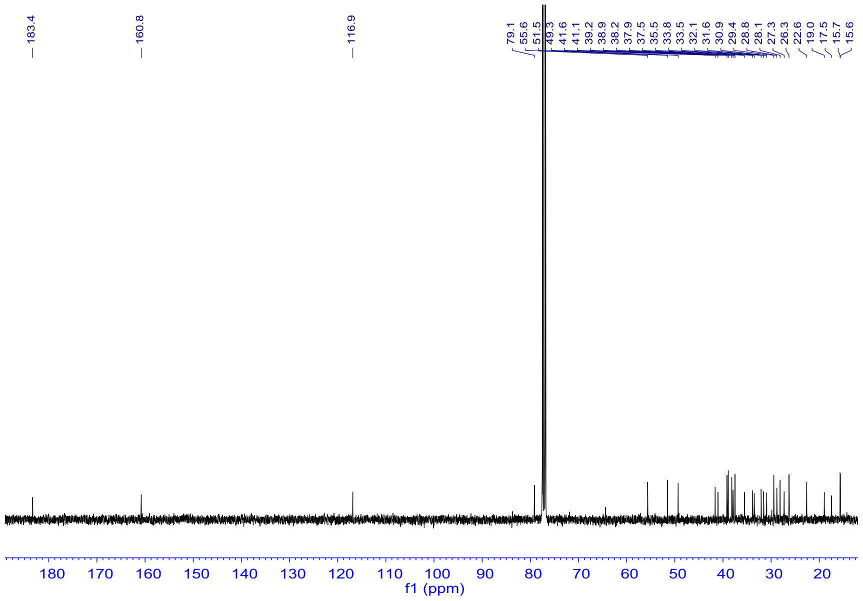

Supplementary Figure 2 13C NMR spectrum of Compound A (100 MHz, CDCl3)

Supplementary reference

[1] Chaudhuri S K, Fullas F, Brown D M, et al. Isolation and structural elucidation of pentacyclic triterpenoids from Maprounea africana[J]. Journal of Natural Products, 1995, 58(1): 1-9.
