# Supplementary material for: Aleuritolic Acid Impaired Autophagic Flux and Induced Apoptosis in Hepatocellular Carcinoma HepG2 Cells
Source: Molecules. 2018 Jun 2;23(6):1338. doi: 10.3390/molecules23061338 (PMC6100546; doi:10.3390/molecules23061338)
Supplement: Supplementary file 1 [file molecules-23-01338-s001.zip › supplementary materials2.pdf]

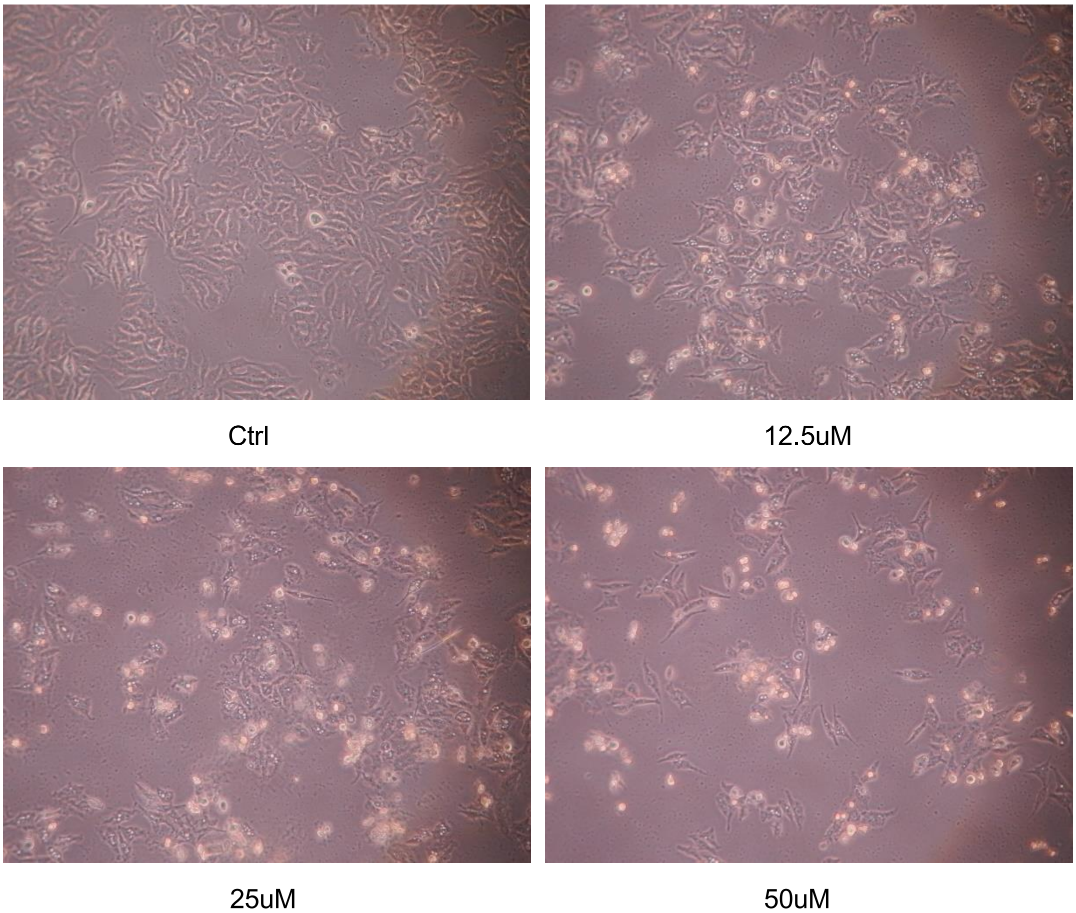

Supplementary Figure 3. Cell density was decreased in a dose-dependent manner and the alterations were comparable with MTT assay. The MTT assay is still reliable.

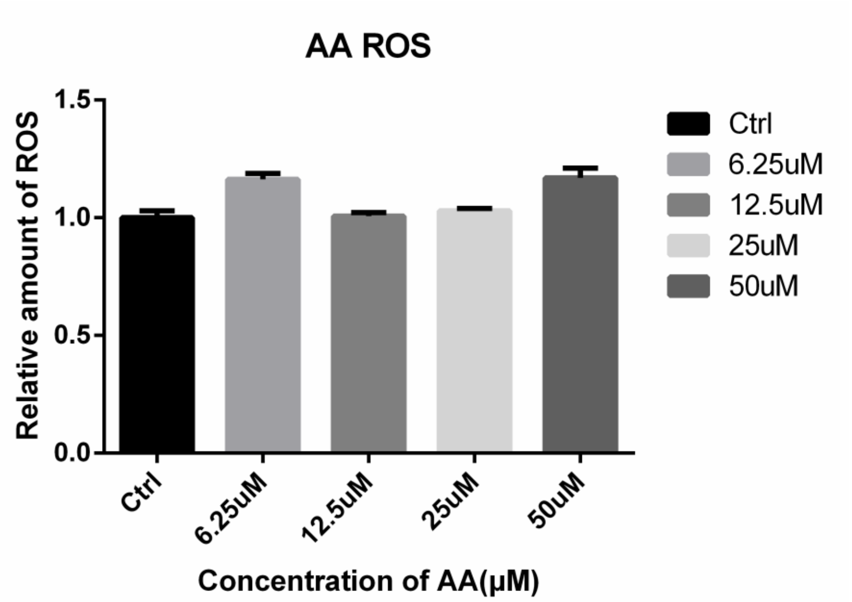

Supplementary Figure 4. Intracellular ROS level was detected using H2DCFDA reagent by flow cytometry. Cells were treated with AA with different concentrations for 24 hours. No trend of enhanced ROS was observed for each contraction tested.
